# Social accountability for reproductive, maternal, newborn, child and adolescent health: A review of reviews

**DOI:** 10.1371/journal.pone.0238776

**Published:** 2020-10-09

**Authors:** Frances Squires, Adriane Martin Hilber, Joanna Paula Cordero, Victoria Boydell, Anayda Portela, Miriam Lewis Sabin, Petrus Steyn

**Affiliations:** 1 Novametrics, Duffield, Derbyshire, United Kingdom; 2 Swiss Centre for International Health, Basel, Switzerland; 3 University of Basel, Basel, Switzerland; 4 UNDP/UNFPA/UNICEF/WHO/World Bank Special Programme of Research, Development and Research Training in Human Reproduction (HRP Research), World Health Organization, Geneva, Switzerland; 5 Global Health Centre, The Graduate Institute Geneva, Geneva, Switzerland; 6 Department of Maternal, Newborn, Child and Adolescent Health and Ageing, World Health Organization, Geneva, Switzerland; 7 The Partnership for Maternal, Newborn, Child & Adolescent Health, Geneva, Switzerland; University of Michigan, UNITED STATES

## Abstract

Globally, increasing efforts have been made to hold duty-bearers to account for their commitments to improve reproductive, maternal, newborn, child and adolescent health (RMNCAH) over the past two decades, including via social accountability approaches: citizen-led, collective processes for holding duty-bearers to account. There have been many individual studies and several reviews of social accountability approaches but the implications of their findings to inform future accountability efforts are not clear. We addressed this gap by conducting a review of reviews in order to summarise the current evidence on social accountability for RMNCAH, identify factors contributing to intermediary outcomes and health impacts, and identify future research and implementation priorities. The review was registered with the International Prospective Register of Systematic Reviews (PROSPERO CRD42019134340). We searched eight databases and systematic review repositories and sought expert recommendations for published and unpublished reviews, with no date or language restrictions. Six reviews were analysed using narrative synthesis: four on accountability or social accountability approaches for RMNCAH, and two specifically examining perinatal mortality audits, from which we extracted information relating to community involvement in audits. Our findings confirmed that there is extensive and growing evidence for social accountability approaches, particularly community monitoring interventions. Few documented social accountability approaches to RMNCAH achieve transformational change by going beyond information-gathering and awareness-raising, and attention to marginalised and vulnerable groups, including adolescents, has not been well documented. Drawing generalisable conclusions about results was difficult, due to inconsistent nomenclature and gaps in reporting, particularly regarding objectives, contexts, and health impacts. Promising approaches for successful social accountability initiatives include careful tailoring to the social and political context, strategic planning, and multi-sectoral/multi-stakeholder approaches. Future primary research could advance the evidence by describing interventions and their results in detail and in their contexts, focusing on factors and processes affecting acceptability, adoption, and effectiveness.

## Introduction

Women, children and adolescents continue to face poor health outcomes, despite the wealth of global strategies, commitments and frameworks that are in place to foster the respect, protection and fulfilment of women’s, children’s and adolescents’ right to health [1]. Ensuring the wellbeing of women, children and adolescents is at the centre of the Sustainable Development Goals, universal health coverage, and a range of United Nations strategies, initiatives, declarations, commitments and guidelines [2–10]. Yet progress at the national level has been uneven, and inequalities in reproductive, maternal, newborn child and adolescent health (RMNCAH) persist [11].

Bridging the gaps between global and national promises and the reality for millions of women, children, and adolescents will, be critical to achieving development goals in the coming decade. Attention to accountability has grown over the last fifteen years, including for women’s, children’s and adolescents’ health, and is increasingly recognised as central to applying human rights to development and health [12].

At its broadest, accountabillity can be defined as “constraints on the exercise of power by external means or internal norms” [13]. Accountability depends on answerability–the obligation to answer questions regarding decisions or actions–and enforceability, i.e. sanctions for illegal or inappropriate actions and behaviour [14]. Importantly, accountability does not just apply to governments, but to anyone wielding power including the private sector, traditional leaders and quasi-state actors [13]. In the case of health systems, accountability lies at the heart of how power relations affect service delivery [15]. Various categorisations of accountability have been described, with one of the most commonly used being: 1) financial accountability, 2) performance accountability and 3) political/democratic accountability [16]. The concept of social accountability falls under this last category, which has to do with ensuring that duty-bearers including governments deliver on their promises, fulfil the public trust, represents citizens’ interests, and respond to societal needs and concerns [16].

Social accountability is gaining acceptance as a way to address health system inefficiencies and improve basic public health performance, including planning and service delivery, and to contribute to the attainment of the highest possible standards of health [17]. Social accountability approaches have been implemented by development agencies, under labels including “citizen participation,” “community engagement,” “citizen demand,” “voice,” “transparency and accountability,” and “good governance” [18]. Like broader accountability initiatives, social accountability may have three types of expected impact: improved quality of governance, increased development effectiveness, and empowerment of disadvantaged or vulnerable groups [19].

The term “social accountability” came into use in the early 2000s to refer to citizen-led processes to demand accountability from governments outside of formal electoral systems [20], and has roots in political science, theories of political administration, and development studies, including rights-based approaches and participatory governance [21]. These diverse origins mean that, while there are some consistent elements, there is no commonly accepted definition of social accountability in the literature. At its core, social accountability refers to “citizens’ efforts at ongoing meaningful collective engagement with public institutions for accountability in the provision of public goods” [20]. The concept encompasses a broad range of actions that citizens, communities, civil society organisations (CSOs), and independent media can use to hold duty-bearers to account [22]. Critical to any social accountability program are opportunities for information exchange, dialogue and negotiation between citizens and duty-bearers; the willingness and ability to seek accountability among citizens and civil society; transparency and open information sharing, attitudes, skills and practices supporting listening and constructive engagement among service providers and policy makers with citizens; and an enabling environment [23,24].

Social accountability approaches are of particular relevance to RMNCAH. The first reason for this is that politics, ideology, and social norms often have a profound impact on both an individual’s realisation of their sexual and reproductive health and rights (SRHR), and their ability to demand accountability [17]. Because accountability is centred on how power is expressed and maintained, political, social, economic and gender relations of unequal power relations are inevitably an issue [13,15]. These power dynamics exert special influence on SRHR, which are inherently bound to social norms around sexuality and gender roles. Secondly, sexual and reproductive health services are beset by accessibility, availability, and quality of care issues, especially for young people and other marginalised and vulnerable groups [25,26].

As a result of the inconsistent terminology used in the social accountability literature, it has been difficult to compare social accountability approaches to draw generalisable conclusions on their impact and effectiveness [27,28]. The same holds for the many individual studies and several reviews examining accountability initiatives in the health sector, some of which have included RMNCAH (Boydell *et al*., 2019 [17]; Lodenstein *et al*., 2017 [21]; Lodenstein *et al*., 2013 [29]; Molyneux, 2012 [30]; McGee, R. Gaventa, 2011 [31]; Boydell and Keesbury, 2014 [32]; Danhoundo, Nasiri and Wiktorowicz, 2018 [33]; Martin Hilber, Blake, *et al*., 2016 [34]; Van Belle *et al*., 2018 [35]). In particular, the relationship between social accountability initiatives and the development, democratic, or empowerment impacts they seek has not been clearly articulated [19]. As a result, there is a lack of clarity about what the evidence for social accountability relating to RMNCAH shows, and questions remain about how, and in what contexts, social accountability efforts can be most effective in achieving their objectives. Calls for more and/or better accountability initiatives do not automatically ensure that future efforts will be appropriate or successful [36].

The objectives of this review are fourfold:

1. To summarise existing reviews of social accountability approaches for RMNCAH, including the characteristics of relevant reviews, the definition of social accountability used, the approaches included, and their objectives, intermediary outcomes and health impacts;
2. To identify factors (e.g. characteristics of the accountability approach, contextual factors) contributing to the intermediary outcomes and health impacts of RMNCAH social accountability initiatives;
3. To determine if there is evidence for the effectiveness of social accountability approaches to address RMNCAH needs, including of marginalised and vulnerable groups;
4. To identify research and implementation priorities for future social accountability initiatives for RMNCAH.

## Methods

We define social accountability broadly as citizen-led, collective processes for holding duty-bearers (including politicians, government officials, and/or service providers) to account for their actions [20,37]. While other definitions of social accountability often include the actions of individuals, we considered only collective or community actions. We consider the efforts of both CSOs, and direct engagement of citizens at the community level, as forms of social accountability. Noting that the over-arching goal of accountability efforts is that authorities are held responsible for executing their powers according to a certain standard, we distinguish social accountability from activism and advocacy by the seeking of a specific action, recourse or remedy from a duty bearer, even if that goal was not achieved.

Social accountability interventions were considered to include: community monitoring, social and community audits, public hearings and community meetings, citizen report cards and community scorecards, verbal and social autopsies, partnership-defined quality, other client feedback mechanisms, citizen-led budget advocacy, and community participation in verification/ validation of data for results-based financing [24,38]. Intermediary outcomes of interest included: community or health care user empowerment, improved health care provider behaviour, health systems strengthening, improvements in service uptake, or changes in legislation, policies, regulations or guidelines. Health impacts of interest included any measure of RMNCAH morbidity or mortality.

We conducted this review according to a peer-reviewed protocol, which was registered with the International Prospective Register of Systematic Reviews (PROSPERO CRD42019134340, https://www.crd.york.ac.uk/prospero/).

### Search and screening strategy

We reviewed published and grey literature, including peer-reviewed journal articles, books, book chapters, electronic articles, reports, and theses. All population and geographic setting were eligible for inclusion, and we imposed no date or language restrictions.

We searched eight databases and systematic review repositories between 14 April and 11 May 2019, including the Centre for Reviews and Dissemination, Cochrane, Global Index Medicus, Health Evidence, PROSPERO, PubMed, Scopus and the International Initiative for Impact Evaluation (3ie). Our search strategy used a wide variety of search terms to produce a high sensitivity search (see Table 1 and S1 Annex). Search terms included combinations of terms related to social accountability, RMNCAH, and review, mapping or synthesis papers and were tailored for each database. We also sought recommendations from a group of 44 social accountability practitioners and academics (the Community of Practice on measuring social accountability and health outcomes, which is a forum for researchers and practitioners undertaking this research and monitoring and evaluation to share experiences, methodologies, and outcomes from their work; and discuss how to action research). Expert recommendations were accepted until 31 August 2019. We examined the bibliographies of included reviews for additional sources.

**Table 1:** Search terms.

Search terms	
Accountability	social accountability; social responsibility; community participation; accountab*; collective action; community action; social mobilisation; social mobilization; community mobilisation; community mobilization; social movement*; community movement*; participatory budgeting; public expenditure tracking; citizen charter*; public hearing*; citizen report card*; social audit*; health committee*; community scorecards; complaint mechanism*; social protest*; participatory governance; social audits; participatory budgeting; patient advocacy; community monitoring
Reproductive, maternal, newborn, child and adolescent health	reproductive health; sexual health; maternal health; newborn health; neonat*; adolescent health; child health; HIV; sexually transmitted disease; STI; gender-based violence; intimate partner violence; violence against women; female genital cutting; female genital mutilation; family planning; contraception; abortion*; cervical cancer
Review, mapping, synthesis	systematic review OR synthes* OR mapping OR review* OR systemat*

### Inclusion and exclusion criteria

We included reviews, mapping exercises, or synthesis studies that had a specific focus on RMNCAH and reviewed at least one social accountability approach. Papers that reviewed other accountability approaches were included as long as they focused on RMNCAH and discussed at least one relevant social accountability approach. Papers that did not primarily focus on RMNCAH were excluded. Reviews on specific interventions, such as mystery client interventions to improve quality of care, which were not clearly labelled as accountability approaches, were excluded. We also excluded primary studies (i.e. those that did not review, map or synthesise other research). Reviews in which the interventions examined did not involve a significant citizen engagement component were not considered relevant, nor were studies of community participation, engagement or mobilisation approaches that did not clearly seek to hold duty-bearers to account (e.g. studies examining village health committees aiming to increase awareness or change behaviour).

**Fig 1:**
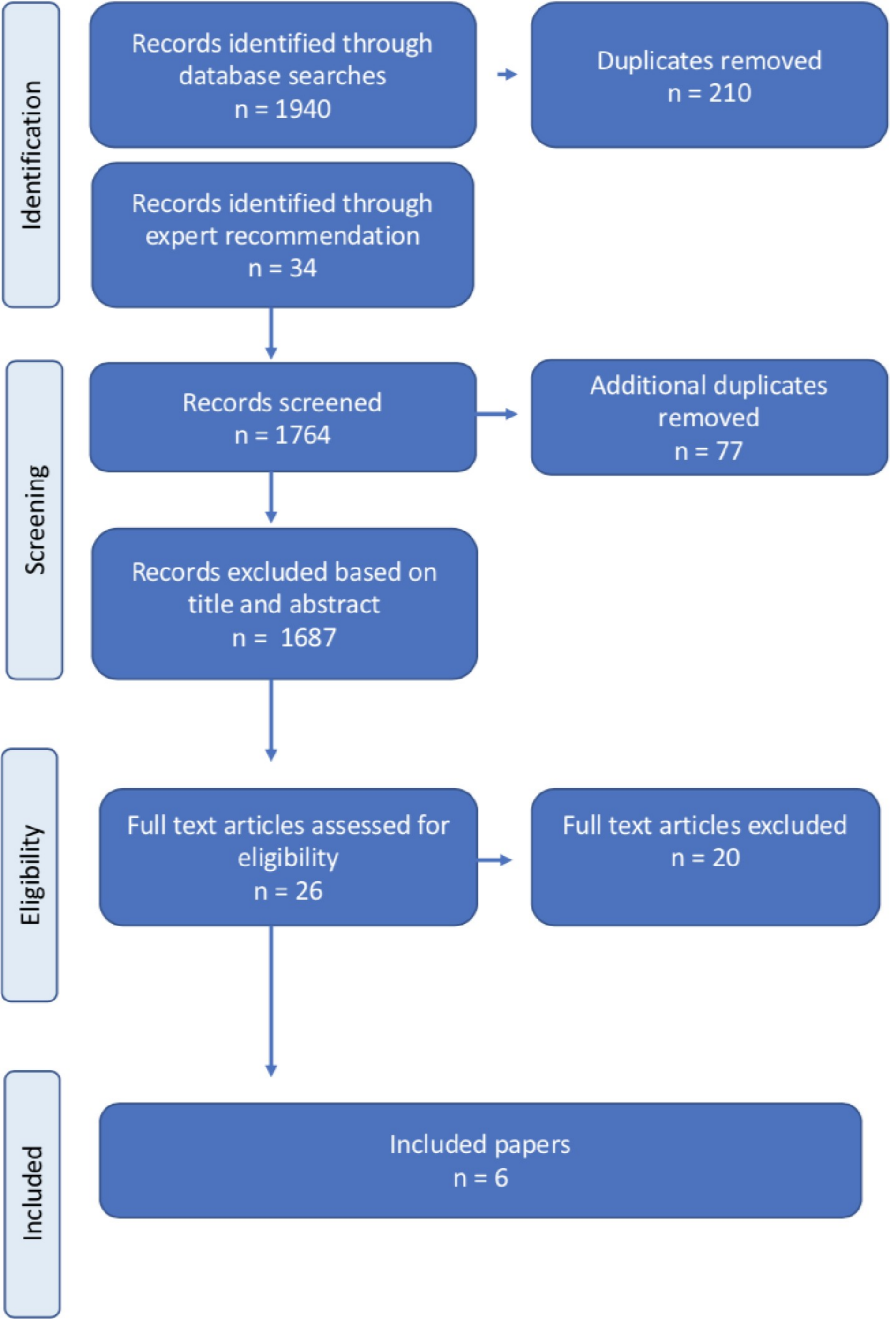
Search flowchart.

Fig 1 shows the search flowchart. Each stage of the search was conducted by at least two reviewers working independently. The titles/abstracts of all search results were reviewed after removal of duplicate results and coded as clearly irrelevant or possibly relevant. The latter group was narrowed to those that were probably or definitely relevant and full texts were obtained. Twenty-six [26] full texts were reviewed and assessed for inclusion by three reviewers (AMH, FS and JC). Differences or uncertainty were resolved through discussion. Twenty [20] papers were excluded at this stage (see S2 Annex); the remaining six articles were abstracted for analysis in duplicate by three reviewers (AMH, FS and JC).

#### Data extraction, analysis and quality assessment

We extracted data on the setting, population, objectives, study design, methodology, and findings from six reviews. For the reviews examining accountability in general, we considered only the findings related to social accountability approaches. For the papers on perinatal mortality audits, we analysed data related to community audits and community involvement in facility-based audits only.

Three reviewers (AMH, FS and JC) assessed each of the six reviews for their equity, social and health impact, and overall quality and reliability using standardised tools. Review quality was assessed using a checklist (S3 Annex), which was developed for this review based on two relevant tools: 3ie's quality appraisal checklist (Snilstveit B, Eyers J et al, 2018), and the GRADE-CERqual approach for assessing confidence of evidence from reviews of qualitative research (Lewin, Booth et al, 2018). Each study was assigned an overall quality assessment of low, medium, or high. Differences between raters’ evaluations were resolved through discussion.

**Fig 2:**
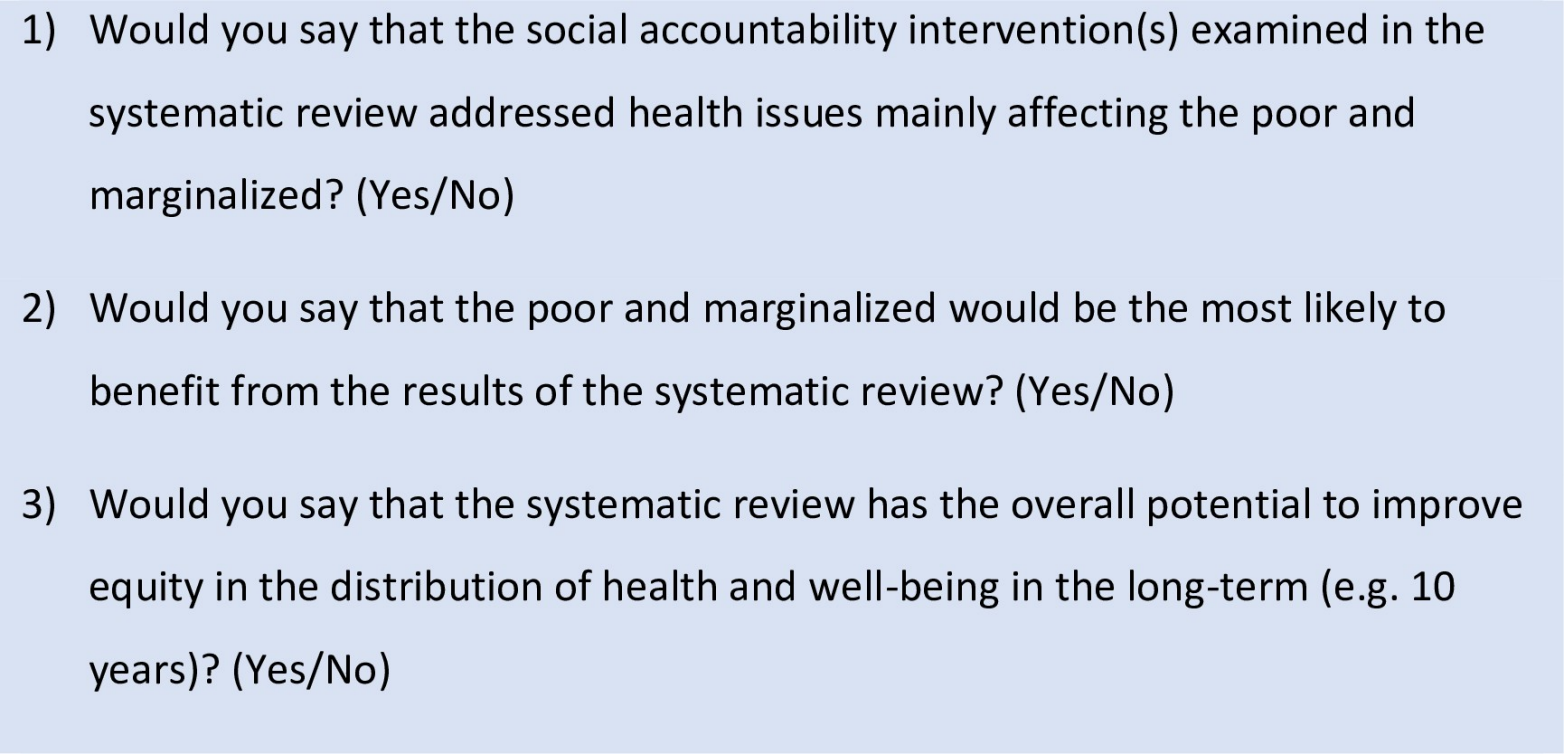
Criteria for evaluating impact on equity.

The reviews were also evaluated for the degree to which they considered equity, using the short checklist shown in Fig 2 [39]. Reviews were rated as high effect (three yes results), medium effect (two yes results), or low effect on equity (one or no yes results) and differences in ratings between reviewers were resolved through discussion.

**Fig 3:**
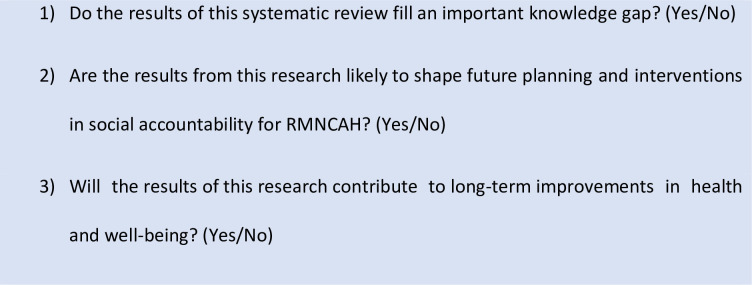
Criteria for evaluating health and social impact.

Like equity, health and social impact were evaluated according to three questions used elsewhere, slightly adapted for our purposes (Sharma, Buccioni et al 2017 [39]; see Fig 3). Ratings were determined in the same manner as described for assessing the impact on equity.

Narrative synthesis was used to summarise extracted data, and thematic analysis was used to examine the different accountability approaches that emerged. We analysed data at the review level only; re-analysis of the primary studies included in the reviews was judged to be beyond the scope of this review. Meta-analysis was not appropriate because of heterogeneity between the included papers, which described a variety of interventions in differing contexts, with limited information about what was done in each setting.

## Results

We retrieved 1974 results and removed 287 duplicates in two stages (see Fig 1). Twenty six full texts were reviewed and 20 of these were excluded; the remaining six articles were extracted for data analysis (Boydell *et al*., 2019 [17]; Boydell and Keesbury, 2014 [32]; Van Belle *et al*., 2018 [35]; Pattinson *et al*., 2016 [40]; Kerber *et al*., 2015 [41]; Martin Hilber, Blake, *et al*., 2016 [42]). A smaller group of articles was excluded because they did not primarily focus on accountability for RMNCAH, but informed our thinking [43–46].

### Review characteristics

The six reviewed articles were: a multi-disciplinary systematic review examining accountability for SRHR globally and a secondary analysis of the results (Boydell *et al*., 2019 [17]; Van Belle *et al*., 2018 [35]), a structured review/mapping investigating accountability for maternal and newborn health in Sub-Saharan Africa (Martin Hilber *et al*., 2016 [42]), and a literature review examining social accountability for family planning and reproductive health [32]. The remaining two articles are a systematic review and a literature review focusing specifically on perinatal mortality audits [40,41]. To clearly distinguish between the two included reviews with Boydell as the first author, we refer to the Boydell *et al*. review from 2019 as Boydell, Schaaf *et al* [17]).

None of the identified reviews focused on other specific social accountability intervention such as participatory budgeting and community score cards. All of the included papers focus entirely or substantially on low and middle income countries. Table 2 shows the design, thematic focus, and objectives of the included reviews.

**Table 2:** Design, thematic focus and objectives of included reviews.

Paper	Review design	Thematic focus	Objectives
Boydell & Keesbury (2014) [32]	Literature review	Social accountability for family planning and reproductive health programs	To synthesise the literature (review papers and individual studies) on social accountability to better understand its potential for improving family planning and reproductive health programs.
Boydell, Schaaf *et al*. (2019) [17]	Secondary analysis of a systematic review (Van Belle *et al*.)	Accountability for sexual and reproductive health and rights	To expand the discussion in Van Belle *et al*.’s review to identify the conditions and processes that are germane to accountability in SRHR, including contextual factors, the interplay between different approaches and processes, and the capability of individuals and communities to negotiate accountability in SRHR.
Kerber *et al*. (2015) [41]	Literature review	Perinatal mortality audit (small subsection on community ownership and partnership)	1. To review national policies and existing national and local systems to assess country progress towards institutionalising facility-based maternal and perina- tal death audit 2. To review the available evidence for perinatal mortality audit and to synthesise the main challenges from the literature within the WHO health system building blocks 3. To propose solutions for scaling up mortality audit for stillbirths and neonatal deaths based on literature and programme learning.
Martin Hilber *et al*. (2016) [34]	Structured review/mapping	Accountability for maternal and and newborn health in Sub-Saharan Africa	To describe the types of maternal and newborn health program accountability mechanisms implemented and evaluated in Sub-Saharan Africa, their effectiveness, and ways to improve governance and maternal and newborn health outcomes.
Pattinson *et al*. (2016) [40]	Systematic review	Perinatal mortality audit (small subsection on perinatal mortality audit at the community level)	To present a systematic review of facility-based perinatal mortality audit in low-and middle-income countries, and review information regarding community audit.
Van Belle *et al*. (2018) [35]	Systematic review	Accountability for sexual and reproductive health and rights	To map the range of accountability strategies and instruments used to address sexual and reproductive health and rights in low and middle income countries, including their contexts and outcomes

The reviews used varying search methodologies and theoretical frameworks for analysis in order to meet their various objectives. Van Belle *et al*. conducted a multi-disciplinary search using health, social science, and law search engines with a realist intent, and used a meta-interpretation approach. Boydell, Schaaf *et al*. conducted a secondary, more detailed thematic analysis of Van Belle et al’s results to explore factors influencing implementation of accountability initiatives for SRHR. Martin Hilber *et al*. conducted a literature search across five academic databases, and used a structured approach based on categories of accountability mechanisms [47]. Boydell and Keesbury considered both peer-reviewed and grey literature to identify results, common trends and thinking in social accountability. The two articles on perinatal mortality audits had narrower objectives, aiming to synthesise the evidence, including information regarding community involvement in audit processes. Both reviews were based on broad searches. Kerber *et al*. structured their analysis using the WHO health system building blocks framework with the addition of community ownership and participation [48] whereas Pattinson *et al*. do not clearly describe their method of analysis for the section on perinatal mortality audit at the community level.

The results of the assessments of review quality, health and social impact, and equity impact are presented in Table 3. Four of the reviews were assessed as low quality, in part due to insufficient richness and quality of evidence in the included studies. The two related reviews by Van Belle *et al*. and Boydell, Schaaf *et al*. were notable for their potential health and social impact and their robust attention to equity.

**Table 3:** Review quality, health and social impact, and equity impact assessment outcomes.

Paper	Review quality	Health and social impact	Equity impact
Boydell & Keesbury (2014) [32]	Low	Moderate	Moderate
Boydell, Schaaf *et al*. (2019) [17]	Medium	High	High
Kerber *et al*. (2015) [41]	Low	Low-moderate	Moderate
Martin Hilber *et al*. (2016) [34]	Low	Moderate	Moderate
Pattinson *et al*. (2016) [40]	Low	Low-moderate	Moderate
Van Belle *et al*. (2018) [35]	Medium	Moderate—high	High

Table 4 shows the number, type and quality of the studies included in each review. The two papers on mortality audits contained scant detail on the number, type and quality of studies included in the sections on community involvement in audits (in contrast with the vast amount of research conducted on facility-based audits). Only Van Belle *et al*. (and by association Boydell, Schaaf *et al*.) provided a detailed description of the types of study included with a rigorous quality assessment of the included studies, though it was noted that the quality of the papers was difficult to assess.

**Table 4:** Number, type and quality of studies included in each review.

Paper	Number of included studies	Type of studies	Quality
Boydell & Keesbury (2014) [32]	29	13 review papers and 16 FP/RH case studies	Not formally assessed. The bulk of the RH/FP experiences were grey literature, primarily of project reports or other deliverables, of varying quality.
Kerber *et al*. (2015) [41]	Unclear for the community audit findings	Unclear for the community audit findings	Unclear for the community audit findings
Martin Hilber *et al*. (2016) [42]	38; five of these related to social accountability for maternal and newborn health	Not reported	Not reported
Pattinson *et al*. (2016) [40]	Unclear for the community audit findings	Unclear for the community audit findings	Unclear for the community audit findings (studies noted to be of low/moderate quality for the overall findings).
Van Belle *et al*. (2018) [35] and Boydell, Schaaf *et al*. (2019) [17]	40; nine of these examined social or community accountability	Systematic review, cross-sectional studies, qualitative case studies, descriptive studies, policy analysis, ethnographies, legal reviews, action research, critical studies, undefined.	Reported as difficult to assess. Eighteen papers presented an audit trail, 15 had a sampling process described, and in 15 papers, triangulation, member checking or deviant case analysis was used to ascertain validity. Fourteen studies obtained the highest score for explanatory power; six obtained the highest score for insider comprehensiveness. Eighteen out of 40 studies displayed some proof of long-term field engagement. Eleven studies clearly distinguished data from interpretation and nine studies displayed some form of reflexivity.

### Findings of the reviews

#### Definitions of accountability and social accountability

The four reviews examining accountability in general use differing definitions and understandings of accountability and social accountability (see Table 5). Although there were common elements, only Boydell and Keesbury include a clear definition of social accountability. Implicit in each review’s discussion (and explicit in the discussion by Van Belle *et al*.) are the power relations between citizens and their governments, which determine whether and how duty-bearers meet their responsibilities to rights-holders.

**Table 5:** Definitions of accountability and related terms.

Paper	Definition
Boydell and Keesbury (2014) [32]	Social accountability is defined as the “efforts of citizens and civil society to scrutinise and hold duty-bearers to account for providing promised services."
Boydell, Schaaf *et al*. (2019) [17]	Accountability describes the processes by which government actors are responsible and answerable for the provision of high-quality and non-discriminatory goods and services (including the regulation of private providers) and the enforcement of sanctions and remedies for failures to meet these obligations.
Kerber *et al*. (2015) [41]	Two definitions of community perinatal audits are described: 1) Surveillance of community births and deaths, in which trained community workers visit households following a death and conduct social and/or verbal autopsy to feed back into a local or centralised data collection system 2) Involvement in facility mortality audit, in which appropriate results are shared and recommendations to address community-related avoidable factors are developed together.
Martin Hilber *et al*. (2016) [42]	Social accountability is recognised as a type of political or democratic accountability, which is defined as “the relationship between the state and the citizen, discussions of governance, increased citizen participation, equity issues, transparency and openness, responsiveness, and trust-building” [16].
Pattinson *et al*. (2016) [40]	Social audit is described as a tool used at community level to identify strategies for community motivation of behaviour change, or for addressing delays and promoting linkages for care. Verbal and social autopsies are defined as tools used in community-level perinatal mortality audit to ascertain the cause of death profile as well as contextual factors related to these deaths.
Van Belle *et al*. (2018) [35]	Accountability for health systems “lies at the heart of how power relations in service delivery are negotiated and implemented.”

Only one definition of accountability (Boydell, Schaaf *et al*.) references the enforcement of sanctions and remedies for failures to meet obligations, although Martin Hilber *et al*. also place significant emphasis on remedy and recourse mechanisms. Boydell and Keesbury noted that none of the reviewed studies addressed the issue of redress or remedy as part of social accountability for family planning and reproductive health programs; rather, papers “emphasise the need to collaborate and support service providers and local officials in place of a more adversarial approach that threatens sanctions and reputational risk.”

The two reviews focusing on mortality audits did not include formal definitions of accountability, since they focused specifically on audits rather than accountability more generally. The definitions they used for community perinatal audit, social audit, and verbal and social autopsy are shown in Table 5.

#### Interventions described in the reviews

Three main groups of social accountability interventions are described by the reviews: 1) accountability through community monitoring of health facilities, including community involvement in perinatal mortality audits; 2) advocacy and activism for greater accountability, often via coordinated efforts by CSOs and/or professional associations; and 3) interventions supported by the development, use, or enforcement of laws, policies, regulations or national monitoring mechanisms.

Most of the social accountability interventions described in reviews include some form of community monitoring of health facilities. Boydell and Keesbury describe nine types of social accountability interventions used for family planning and reproductive health services, of which public expenditure tracking, citizen report cards, social audits, community scorecards, health committees, and information sharing or campaigns were most commonly used. Two of the five articles on social accountability reviewed by Martin Hilber *et al*. examined the role of communities in improving the quality of services and accountability of health providers and local government, with interventions including health facility committees, community participation in local government processes (including tracking of progress on commitments), and participatory scorecards to monitor health services and/or improve quality of care. Boydell, Schaaf *et al*. and Van Belle *et al*. also identified monitoring interventions, including stakeholder meetings, public hearings, community scorecards and dashboards, health committees, and citizen charters. Of the community monitoring interventions, the review findings suggest that evidence is strongest for community scorecards, citizen charters, and health committees, which have been shown to contribute to several intermediary outcomes. One example was a landmark randomised controlled trial which documented increases in service utilisation and improvements in child mortality and child weight a year after the implementation of a community monitoring intervention involving report cards [49].

Similarly, the two reviews focusing on perinatal mortality audit describe how community involvement in audit processes can contribute to actions at the heath service-level to improve quality of care. Kerber *et al*. describe two main ways of facilitating community ownership and partnership in perinatal mortality audits: 1) community engagement to capture births, deaths and associated factors at the community level, and 2) community participation in facility-based death reviews. These approaches aim to integrate community ownership and partnership into a national audit system, by identifying strategies for community motivation of behaviour change, and/or addressing delays and promoting linkages for care. Though community involvement in audit processes does not always explicitly contribute to greater accountability, community involvement in audit processes could be considered both a community monitoring tool and a way of building partnership between health services and the communities they serve, strengthening data collection, and addressing community or context-related factors that result in poor health outcomes. The papers give examples of community audit interventions from India, Indonesia, Guinea, Malawi, and Uganda, ranging from using a local music and drama troupe to facilitate community meetings, to community maternal and perinatal mortality audits as part of a district-based strategy, and verbal and social autopsies.

The second group of interventions involves CSOs, health service users, community groups and/or activists working together in calling for accountability for RMNCAH from governments. For example, Martin Hilber *et al*. reported on how partnerships between community groups and professional associations were used to advocate for health systems strengthening to reduce maternal mortality, based on the South African Treatment Action Campaign model [50].

The third group of interventions relates to social accountability mechanisms supported by laws, policies, regulations, or monitoring bodies. This group largely describe the efforts of CSOs, rather than the direct engagement of citizens at the community level. For example, Van Belle *et al*. documented how national policies and legal systems play a role in delivering accountability, including the role of CSOs in preparing or bringing cases on SRHR violations before a court. In specific examples, a court in the Indian state of Madhya Pradesh ordered the immediate implementation of maternal death audits [51] and gender laws in Nepal and Sri Lanka were modified as a result of civil society demands [52]. Martin Hilber *et al*. also documented the use of national-level social accountability mechanisms, such as an Independent Accountability Mechanism (NIAM) run by an independent civil society-led Expert Review Group (iERG) in Nigeria to track MNH commitments and action plans [53].

There are significant overlaps and interactions between the three groups, with different types of interventions used in different ways and to achieve different objectives. For example, interventions designed to empower health services users to demand better healthcare via community monitoring tools such as scorecards, may result in communities forming partnerships to advocate for legal or policy changes. Though evidence on how and why particular interventions have been implemented in specific contexts to achieve certain objectives is lacking, Van Belle *et al*. noted that strategies to improve service delivery, including community monitoring mechanisms, tend to focus on maternal, neonatal and child health, whereas legal and policy activism generally addresses accountability for HIV, gender-based violence and lesbian, gay, bisexual, and transgender (LGBT) concerns.

#### Intermediary outcomes and health impacts

The results of social accountability interventions for RMNCAH have not been well documented, due to the design of both the reviews and the studies they included. The reviews included here all have a bias towards published, peer-reviewed literature, which means they represent a small proportion of social accountability efforts, and may have missed learnings from community-initiated approaches or project-specific accountability efforts. Additionally, the results of social accountability initiatives are difficult to compare, due to the diverse methods used to study them, and lack of clarity regarding assumptions about inputs and outcomes [32].

Nevertheless, many studies included in the reviews report positive findings regarding the potential of social accountability interventions to achieve intermediary outcomes, and in some cases, health impacts. Reviews tend to focus on intermediary outcomes more than health impacts, which are more difficult to demonstrate. Boydell and Keesbury did not report specific findings on the impact of the interventions assessed due to limitations in the evidence but noted that half the case studies they reviewed reported positive outcomes, including increased funding, increased rates and timeliness of health care seeking, decreased maternal deaths, and improved staffing at healthcare facilities. Van Belle *et al*. noted that although the reviewed studies reported on several types of intermediary outcomes, including the strengthened capacities of rights holders, provider practices, health system level outcomes, and changes in policy, legislation or guidelines, few were able to document health impacts such as reduced mortality. Boydell, Schaaf *et al*. expanded on this by commenting that currently, accountability efforts appear to focus on awareness-raising and increasing knowledge of entitlements over transformational change in norms within health systems and communities.

Reported benefits of community involvement in the audit cycle included avoiding incorrect transfer of blame to the first delay (decision to seek care) and other community-related factors; community education, empowerment and attitude or behaviour change (e.g., reduced fatalism around poor neonatal health outcomes); and improvements to health services, such as better supply of drugs and equipment [54]. No evidence of health impacts as a result of community involvement in audits was presented.

#### Factors influencing successful implementation of social accountability interventions

The reviews note several factors that influence the success of social accountability interventions for RMNCAH.

Van Belle *et al*. reported on contextual conditions that influenced the success of accountability interventions, including broad social structures, governance factors, and core features of the health system. However, few of the contextual descriptions in the included studies were detailed, and analysis of the contribution of contextual factors to the outcomes or health impacts was often lacking. Boydell, Schaaf *et al*. expanded on this by discussing the strong influence of prevailing politics and ideologies, health system responsiveness, and the complex nature of health systems as key factors affecting the successful implementation of accountability interventions.

Both Martin Hilber *et al*. and Boydell, Schaaf *et al*. commented on the importance of multi-stakeholder and multi-sectoral approaches, although few such efforts were identified in the reviewed evidence. Boydell, Schaaf *et al*. noted that interventions working at multiple levels and across different parts of the health system’s “web of accountability” are more likely to achieve change in a complex, adaptive system. This is of particular relevance with regard to SRHR, where ideological and political determinants play a significant role. Indeed, Martin Hilber *et al*. reported that multi-stakeholder and multi-tactic approaches that target underlying norms affecting the determinants of health, as well as, specific problems such as the quality of health care available, supported by laws, policies, or international human rights obligations have had the greatest impact in Sub-Saharan Africa.

Combining these two factors (tailoring to the context and multi-sectoral approaches) by planning strategically according to the intended objectives and the context in order to implement a well-designed, multi-sectoral approach seems to be critical for success. Boydell and Keesbury describe the importance of a clearly articulated theory of change to interrogate the implicit assumptions about inputs and expected results of social accountability initiatives. As noted by Van Belle *et al*., the success of accountability strategies is influenced both by context-specific factors, and “the ability of community to negotiate accountability.” Boydell, Schaaf *et al*. also note that successful accountability efforts work on multiple levels across the health, education, social protection and human rights sectors, adding that such approaches are particularly important to SRHR, where ideological and political determinants contribute significantly to problems with service delivery.

The two reviews examining perinatal mortality audit describe challenges in facilitating community involvement in audits, which echo the above findings. Key challenges include implementation difficulties related to the presence of multiple role-players, the risk of alienating respondents, and the need to counter prevailing power dynamics and social inequalities in order to achieve a valid representation of the barriers to seeking and accessing facility-based care.

#### Review findings on adolescents and other marginalised groups

The reviews give differing levels of attention to adolescents and other marginalised or vulnerable groups, but present scant evidence on social accountability interventions for these groups specifically. The two papers on perinatal/maternal mortality audit do not present any findings on equity or specifically related to adolescent mothers or other marginalised or vulnerable groups in the sections on community involvement in the audit cycle. Martin Hilber *et al*. discuss human rights-based approaches to accountability, informed by efforts to improve access to treatment for prevention of mother-to-child transmission of HIV in South Africa, but did not find other specific references to marginalised or vulnerable groups within the studies reviewed. Similarly, Boydell and Keesbury noted that although some studies reflected that the clients of family planning and reproductive health services may be marginalised, and that there is a need to protect their rights and disaggregate data when implementing social accountability initiatives, they did not present detailed findings on marginalised or vulnerable groups.

Van Belle *et al*. systematically assessed whether the included studies reported on outcomes related to equity, finding that few did so. Though several studies reportedly focused on marginalised groups or communities, or commented on their potential to positively influence equity, the authors note that social inclusion and legitimate representation of marginalised groups were not necessarily achieved. The experiences of adolescents and sex workers were not reflected in the included studies, and no studies examined how accountability strategies can address structural inequalities or benefits distribution across populations.

Boydell, Schaaf *et al*. frame their discussion firmly in recognition that norms, values, bias and stigma heavily influence both the ability of citizens to demand better SRHR, and the likelihood that those in positions of power will respond to their demands. They note that political, religious and cultural ideologies about gender, sexuality and reproduction can affect social practices and the health system, skewing resource allocations or limiting the autonomy of adolescent girls and women. The authors conclude that efforts are needed to overcome norms, values, bias and stigma that discourage individuals from demanding better SRHR and that limit those in positions of power from responding.

#### Evidence gaps

Several gaps in the evidence on social accountability for RMNCAH are notable. In terms of documentation and description, the reviews provide little detail on the genesis and objectives of the social accountability interventions, which stakeholders were involved, the mechanism by which greater accountability will be achieved (e.g., how calling duty-bearers to account will result in greater accountability through specific mechanisms such as action and reporting feedback loops), or how improved accountability can be sustained, institutionalised, or scaled up. Some specific accountability mechanisms are also less well described than others, including participatory budgeting, and the role of parliaments, elections and protest actions (Van Belle *et al*.*)*. Accountability initiatives to address specific areas of RMNCAH have also not been reported on in the accountability literature, including safe abortion and reproductive cancers (Van Belle *et al*.*)*, and studies on social accountability approaches to RMNCAH in humanitarian settings are lacking. Further, Boydell, Schaaf *et al*. note that the impact of gender, stigma, collective struggle, social risk, human rights, and conscientisation are not extensively explored in the literature on accountability for health, although there is a significant literature within HIV and other fields on this.

The unintended effects of social accountability interventions have also rarely been documented, and little published evidence addresses the issue of redress or remedy as part of social accountability for RMNCAH. Rather, as noted by Boydell and Keesbury, papers emphasise the need “to collaborate and support service providers and local officials in place of a more adversarial approach that threatens sanctions and reputational risk” [32].

## Discussion

This review of reviews has confirmed that there is an extensive and steadily growing body of evidence around social accountability in RMNCAH. It is clear that a range of individual interventions has some evidence of promising results. This is particularly true for community monitoring approaches, which have been shown to contribute to outcomes including increased funding, improved staffing at healthcare facilities, and changes in policy, legislation and guidelines, and awareness-raising among rights holders, among others.

It is difficult to draw firm conclusions, however, from the existing evidence about the intermediary outcomes and health impacts of social accountability approaches. Two main factors contribute to this problem. Firstly, reviews of social accountability approaches must overcome inherent difficulties in drawing direct comparisons between studies, both because social accountability approaches are fundamentally community- and context-specific, and because a lack of clarity on key definitions means that any review of “social accountability” gathers information relating to a highly diverse group of interventions. Secondly, a paucity of well-conducted individual studies with clearly articulated objectives, interventions and results limit the available evidence. There are also significant gaps in documentation, particularly relating to contextual detail, intermediary outcomes, health impacts, and unintended effects.

Indeed, few social accountability interventions for RMNCAH have well-documented theories of change to describe their intended objectives, causal pathways, and underlying assumptions. Assumptions about the expected outcomes and how they will be achieved remain implicit. It appears, for example, that community monitoring interventions such as scorecards are more frequently used with the aim of improving quality of care in local health services, whereas community coalitions are more often used to advocate for increased funding or changes in policies or legislation. Because these assumptions have not yet been clearly articulated in the published evidence, or tested via well-designed, documented and evaluated interventions, we cannot know whether they hold true, and if so, in what contexts. It is also important to note that the intended aims of social accountability approaches–and therefore the assessment of whether they have been achieved–may vary according to who defines and evaluates them, but these issues have not been explored in detail in the existing documentation.

As noted by Boydell, Schaaf *et al*., few social accountability interventions documented within the RMNCAH field to date focus on institutionalising change in health systems, on changing community norms, or addressing human rights violations. The majority of the documentation focuses on monitoring of health services through health facility committees, citizen hearings, councils or oversight bodies, rather than on multi-sectoral approaches that address complex contextual factors such as health system responsiveness or restrictive social and gender norms. Most documented accountability interventions involve collection and sharing of relevant information, a discussion between health service users and providers, and in some cases, shared development and implementation of an action plan to address community concerns. While these interventions monitor, review, and in some cases, act on a problem, the documented evidence for their ability to achieve systemic change is limited. This gap in evidence does not necessarily imply that social accountability approaches cannot achieve transformational, systemic change; rather, it probably arises from a combination of factors including the lack of clear articulation of the objectives of social accountability interventions for RMNCAH, the inherent complexity of measuring (and attributing) transformational change, and the long-term timeframes needed to explore systemic outcomes.

Our findings demonstrate other gaps in the documented evidence on social accountability approaches for RMNCAH. Interventions aiming to influence accountability for RMNCAH are complicated by the multiple hierarchies of supervision, management and bureaucracy that circumscribe the accountability “ecosystem” [17,35,55,56]. Few studies have investigated in detail how differing but complementary “types” of accountability interventions–e.g. performance accountability, financial accountability, and social accountability–can interact across the health system, in terms of both intended and unintended effects. Furthermore, attention to how marginalised and vulnerable groups can best be supported to claim their rights, and challenge violations of their rights and factors that give rise to unacceptable inequity, is largely missing from current evidence. This issue has recently garnered increasing attention, for example at the Community of Practitioners on Accountability and Social Action in Health (COPASAH) meeting in 2019 and at the WHO Community of Practice on measuring social accountability the same year. Given the critical importance to SRHR of social, political, religious and cultural ideologies, including gender norms, addressing this gap should be a priority for future research. Similarly, as increasing numbers of people globally endure humanitarian crises [57], it will be important to explore how social accountability approaches can work in humanitarian settings.

Despite the challenges of researching and documenting social accountability, our review identified some factors that may increase the likelihood of an intervention successfully achieving its objectives. Social accountability efforts are more effective when interventions are tailored to the historical, social, cultural, economic, political and moral context, taking into account community priorities, health system complexities, and local political and social ideologies. The use of strategic, multi-sectoral approaches that build links between complementary accountability mechanisms, can also contribute to success [28]. The Treatment Action Campaign in South Africa was cited as a strong example of what can be achieved via collective action to engage the legal, policy, and human rights sectors, as well as professional associations and citizens (Martin Hilber, Blake, *et al*., 2016 [42]; Ray, Madzimbamuto and Fonn, 2012 [50]). Finally, linkages to redress and remedy mechanisms are often overlooked by social accountability approaches. Social accountability efforts for RMNCAH rarely define what recourse is needed if duty-bearers fail to meet their responsibilities, and may benefit from learnings from other fields of literature on this, including evidence related to legal empowerment [20,58,59].

Our findings are supported by literature from outside the social accountability and RMNCAH fields. There are several well-conducted reviews of community engagement, mobilisation, and participation, as well as human rights-based approaches to RMNCAH, which have significant overlap with accountability approaches [43–46,60–62]. Similarly, findings on accountability initiatives from outside the RMNCAH field can enhance our understanding of effective approaches that could be applied to RMNCAH [23,29–31,33,36,63–68]. In particular, lessons could be drawn on the conceptualisation and measurement of “empowerment,” which though often listed as an individual or collective outcome of social accountability approaches, appears to be infrequently operationalized, and poorly described in existing research [69,70]. The significant crossover between these related fields serves as a reminder that social accountability in health is fundamentally a collective call for action on a community concern, and therefore is most effective when efforts are driven by those with a stake in the outcome. Social accountability efforts should, therefore, support community members, particularly those most marginalised, with tools, approaches, training and facilitation to voice their concerns and seek solutions. While a community may or may not aim to (or succeed in) holding someone to account for a health systems issue, the very act of engaging can achieve benefits, including individual and collective empowerment, voice, and community cohesion.

Future primary research efforts on social accountability approaches to RMNCAH should be targeted to address gaps in documentation, including the political factors, processes and incentives that mediate the adoption, effectiveness and acceptability of accountability initiatives, and the national governance reforms and contexts that can enhance accountability [71]. The current evidence base has been limited not by a lack of experience on the positive effects of collective action for change, but because the research and documentation methods used to date have not captured the full breadth and depth of efforts to build social accountability for RMNCAH. Traditional study designs often struggle to capture the underlying context, empowerment and community engagement processes and outcomes, and to isolate the multifaceted (intended and unintended) effects social accountability approaches may have across a complex health system [72,73]. More holistic and complexity-informed primary research is needed, to develop rich information on how interventions were designed and implemented for their specific contexts, and their results. In particular, efforts are needed to develop and describe theories of change underlying social accountability approaches, to better articulate social accountability definitions, causal pathways to change, and underlying assumptions. Furthermore, future reviews on this topic may be strengthened by focussing on sub-topics, for example particular groups of similar interventions, in order to allow more in-depth analysis of findings. Learnings from relevant recent realist and in-depth narrative reviews [63,64], and an upcoming systematic review of study designs to measure effects of social accountability interventions on RMNCAH programs [74], could be used to inform future research.

This review of reviews is the first to systematically analyse reviews of social accountability approaches for RMNCAH, and provides a useful overview of what evidence has been documented in this field, as well as identifying priorities for ongoing research. However, our search only captured peer-reviewed literature, and is likely to have missed some learnings, especially relating to community-initiated initiatives. Additionally, lack of consistency in nomenclature, and the overlap and interactions between related areas including community mobilisation, participation, engagement and empowerment, meant that it was difficult to search precisely for articles on social accountability, or to extract the relevant findings from papers on related topics. Furthermore, the lack of detail in descriptions of interventions at both the study and review level posed challenges for understanding and assessing what has been done, by and for whom, for what objectives, and with what results, in particular contexts. This prevents us from drawing conclusions about which interventions should be prioritised in order to achieve desired intermediary outcomes and health impacts in differing contexts, and beyond—to bring about systemic and transformational change in power relations.

## Conclusion

At a recent international meeting on social accountability, an activist from an indigenous community in Central America was asked how her community had sustained their social accountability campaign over several years. She responded, “We have no choice. It is our health and lives at stake” (The Partnership for Maternal, Newborn and Child Health, 2018) [75].

Our review echoes her words: the health of individuals and communities must be firmly at the heart of social accountability efforts for RMNCAH. Our findings confirm the promise of social accountability to improve the health and lives of women, children and adolescents, despite limitations and gaps in the current documented evidence. Carefully-designed, strategic, multi-stakeholder approaches, tailored to the community’s context, offer the greatest potential for lasting transformational change for RMNCAH. Careful effort to address gaps in documentation with well-designed studies that clearly articulate their objectives and theories of change (including assumptions in causal pathways) is needed to ensure learnings are captured for future accountability initiatives, both within and beyond the field of RMNCAH.

## Supporting information

S1 File PRISMA 2009 checklist—SA review 22.04.20. (DOC)

S2 File SA review—final combined search results. (XLSM)

S1 Annex PubMed search strategy. (DOCX)

S2 Annex Articles excluded at full text review. (DOCX)

S3 Annex Quality assessment checklist. (DOCX)

## References

[1] United Nations Economic and Social Council. Special edition: progress towards the Sustainable Development Goals: Report of the Secretary-General. 2019.

[2] World Health Organization. Universal Health Coverage (UHC) [Internet]. 2019 [cited 2019 Sep 22]. Available from: https://www.who.int/en/news-room/fact-sheets/detail/universal-health-coverage-(uhc).

[3] United Nations Department of Economic and Social Affairs. Sustainable Development Goals Knowledge Platform. [Internet]. 20p19 [cited 2019 Sep 22]. Available from: https://sustainabledevelopment.un.org.

[4] Starrs A. The Safe Motherhood action agenda: priorities for the next decade. Report on the Safe Motherhood Technical Consultation, 18–23 October 1997, Colombo, Sri Lanka. New York; 1997.

[5] United Nations. Every Woman Every Child. The Global Strategy for Women’s and Children’s Health (2016–2030). Survive, Thrive, Transform. New York; 2015.

[6] United Nations. International Conference on Population and Development Program of Action. New York: United Nations Population Fund; 1994.

[7] United Nations Economic and Social Council. General Comment No. 14: The Right to the Highest Attainable Standard of Health (Art. 12 of the Covenant). Geneva; 2000.

[8] United Nations Human Rights Council. Technical guidance on the application of a human-rights based approach to the implementation of policies and programmes to reduce preventable maternal morbidity and mortality. New York: United Nations; 2012.

[9] United Nations Human Rights Council. United Nations Human Rights Council. Resolution of the United Nations Human Rights Council on preventable maternal mortality and morbidity and human rights. New York: United Nations; 2012.

[10] United Nations Human Rights Council. Preventable maternal mortality and morbidity and human rights 11 ed. New York; 2009.

[11] World Health Organization. State of inequality: reproductive, maternal, newborn and child health. 2015.

[12] United Nations Secretary General Ban Ki-moon. Global Strategy for Women’s and Children’s Health. Health (San Francisco). 2010.

[13] Freedman LP, Schaaf M. Act global, but think local: Accountability at the frontlines. Reprod Health Matters. 2013;21(42):103–12. 10.1016/S0968-8080(13)42744-1 24315067

[14] Schedler A, Diamond L, Plattner MF, editors. Conceptualising accountability In: The Self-Restraining State: Power and Accountability in New Democracies. London: Lynne Reinner; 1999.

[15] George A. Accountability in health services: transforming relationships and contexts. Vol. 13 2003.

[16] Brinkerhoff DW. Accountability and health systems: toward conceptual clarity and policy relevance. Health Policy Plan. 2004;19(6):371–9. 10.1093/heapol/czh052 15459162

[17] Boydell V, Schaaf M, George A, Brinkerhoff DW, Van Belle S, Khosla R. Building a transformative agenda for accountability in SRHR: lessons learned from SRHR and accountability literatures. Sex Reprod Heal Matters. 2019;27(2):1622357. 10.1080/26410397.2019.1622357 PMC7942763 31533591

[18] O’Meally SC. Mapping Context for Social Accountability: A resource paper. Washington D.C.; 2013.

[19] Gaventa J, Mcgee R. The impact of transparency and accountability initiatives. Dev Policy Rev. 2013;31(S1):S3–28.

[20] Joshi A. Legal Empowerment and Social Accountability: Complementary Strategies Toward Rights-based Development in Health? World Dev. 2017;99:160–72.

[21] Lodenstein E, Dieleman M, Gerretson B, Broerse JE. Health provider responsiveness to social accountability initiatives in low- and middle-income countries: A realist review. Health Policy Plan. 2017;32:125–40. 10.1093/heapol/czw089 27375128

[22] Malena C, Forster R, Singh J. Social Accountability: An Introduction to the Concept and Emerging Practice. Soc Dev Pap Particip Civ Engagem [Internet]. 2004;(76). Available from: http://siteresources.worldbank.org/INTPCENG/214578-1116499844371/20524122/310420PAPER0So1ity0SDP0Civic0no1076.pdf.

[23] Hoffman KD. The role of social accountability in improving health outcomes: Overview and analysis of selected international NGO experiences to advance the field. Washington D.C: CORE Group; 2014.

[24] World Bank. Social accountability sourcebook. Washington, DC: World Bank; 2002.

[25] Mazur A, Brindis CD, Decker MJ. Assessing youth-friendly sexual and reproductive health services: A systematic review. BMC Health Serv Res. 2018;18(1):1–12. 10.1186/s12913-017-2770-6 29587727 PMC5870922

[26] Singh NS, Smith J, Aryasinghe S, Khosla R, Say L, Blanchet K. Evaluating the effectiveness of sexual and reproductive health services during humanitarian crises: A systematic review. PLoS One. 2018;13(7):1–19. 10.1371/journal.pone.0199300 PMC6035047 29980147

[27] Ringold D, Holla A, Koziol M, Srinivasan S. Citizens and service delivery: Assessing the use of social accountability approaches in human development. Citizens and Service Delivery. Washington, D.C.; 2011.

[28] Fox JA. Social Accountability: What Does the Evidence Really Say? World Dev [Internet]. 2015;72:346–61. Available from: 10.1016/j.worlddev.2015.03.011.

[29] Lodenstein E, Dieleman M, Gerretson B, Broerse JE. A realist synthesis of the effect of social accountability interventions on health service providers’ and policymakers’ responsiveness. Syst Rev. 2013;2:98 10.1186/2046-4053-2-98 24199936 PMC4226265

[30] Molyneux S et al Community accountability at peripheral health facilities: a review of the empirical literature and development of a conceptual framework. Heal policy planningPlan. 2012;27(7):541–54. 10.1093/heapol/czr083 PMC3465752 22279082

[31] McGee R. Gaventa J. Shifting Power? Assessing the Impact of Transparency and Accountability Initiatives. [Internet]. 2011 Available from: http://www.ids.ac.uk/files/dmfile/Wp383.pdf.

[32] Boydell V, Keesbury J. Social accountability: What are the lessons for improving family planning and reproductive health programs? A review of the literature Working paper. Washington D.C: Population Council; 2014.

[33] Danhoundo G, Nasiri K, Wiktorowicz ME. Improving social accountability processes in the health sector in sub-Saharan Africa: A systematic review. BMC Public Health. 2018;18(1):1–8. 10.1186/s12889-018-5407-8 PMC5899409 29653531

[34] Martin Hilber A, Blake C, Bohle LF, Bandali S, Agbon E, Hulton L. Strengthening accountability for improved maternal and newborn health: A mapping of studies in Sub-Saharan Africa. Int J Gynaecol Obstet. 2016 12;135(3):345–57. 10.1016/j.ijgo.2016.09.008 27802869

[35] Van Belle S, Boydell V, George AS, Brinkerhoff DW, Khosla R. Broadening understanding of accountability ecosystems in sexual and reproductive health and rights: A systematic review. PLoS One. 2018;13(5):e0196788 10.1371/journal.pone.0196788 29851951 PMC5978882

[36] Brinkerhoff DW, Jacobstein D, Kanthor J, Rajan D, Shepard K. Accountability, health governance, and health systems: Uncovering the linkages: Marshalling the evidence for health governance thematic working group report [Internet]. Health Finance & Governance project; 2017 Available from: https://www.hfgproject.org/accountability-health-governance-health-systems-uncovering-linkages/.

[37] Joshi A, Houtzager PP. Widgets or Watchdogs? Conceptual explorations in social accountability. Public Manag Rev. 2012;14(2 Special Issue: The Politics and Governance of Public Services in Developing Countries):145–62.

[38] World Health Organization and International Initiative for Impact Evaluation. An evidence map of social, behavioural and community engagement interventions for reproductive, maternal, newborn and child health. Geneva; 2017.

[39] Sharma R, Buccioni M, Gaffey MF, Mansoor O, Scott H BZ. Setting an implementation research agenda for Canadian investments in global maternal, newborn, child and adolescent health: a research prioritization exercise. C Open. 2017;5(1):E82–9. 10.9778/cmajo.20160088 PMC5378526 28401123

[40] Pattinson R, Kerber K, Waiswa P, Day LT, Mussell F, Asiruddin S, et al Perinatal mortality audit: Counting, accountability, and overcoming challenges in scaling up in low‐ and middle‐income countries. Int J Gynaecol Obstet. 2016;107. 10.1016/j.ijgo.2009.07.011 19815206

[41] Kerber KJ, Mathai M, Lewis G, Flenady V, Erwich JJ, Segun T, et al Counting every stillbirth and neonatal death through mortality audit to improve quality of care for every pregnant woman and her baby. BMC Pregnancy Childbirth. 2015/09/24. 2015;15 Suppl 2:S9. 26391558 10.1186/1471-2393-15-S2-S9 PMC4577789

[42] Martin Hilber A, Blake C, Bohle LF, Bandali S, Agbon E, Hulton L. Strengthening accountability for improved maternal and newborn health: A mapping of studies in Sub-Saharan Africa. Int J Gynecol Obstet. 2016;135(3):345–57. 10.1016/j.ijgo.2016.09.008 27802869

[43] Ferguson L, Halliday E. Women’s and Children’s Health: Evidence of Impact of Human Rights In: Bustreo Flavia, Hunt P, editor. Women and Children’s Health: Evidence of Impact of Human Rights. Geneva: World Health Organization; 2013 p. 68–81.

[44] George AS, Branchini C, Portela A. Do Interventions that Promote Awareness of Rights Increase Use of Maternity Care Services? A Systematic Review. PLoS One. 2015;10(10):e0138116 10.1371/journal.pone.0138116 26444291 PMC4596618

[45] Gulaid LA, Kiragu K. Lessons learnt from promising practices in community engagement for the elimination of new HIV infections in children by 2015 and keeping their mothers alive: summary of a desk review. J Int AIDS Soc. 2012 7;15 Suppl 2:17390. 22789645 10.7448/IAS.15.4.17390 PMC3499909

[46] Steyn PS, Cordero JP, Gichangi P, Smit JA, Nkole T, Kiarie J, et al Participatory approaches involving community and healthcare providers in family planning/contraceptive information and service provision: a scoping review. Reprod Health. 2016 7;13(1):88 10.1186/s12978-016-0198-9 27449128 PMC4957852

[47] Brinkerhoff D. Taking account of accountability: A conceptual overview and strategic options. Washington DC: USAID; 2001.

[48] World Health Organization. Everybody’s business–strengthening health systems to improve health outcomes: WHO’s framework for action. Geneva: World Health Organization; 2007.

[49] Björkman M, Svensson J. Power to the People: Evidence from a Randomized Field Experiment on Community-Based Monitoring in Uganda. Q J Econ. 2009;124(2):735–769.

[50] Ray S, Madzimbamuto F, Fonn S. Activism: working to reduce maternal mortality through civil society and health professional alliances in sub-Saharan Africa. Reprod Health Matters. 2012;20(39):40–9. 10.1016/S0968-8080(12)39617-1 22789081

[51] Kaur J. The role of litigation in ensuring women’s reproductive rights: an analysis of the Shanti Devi judgement in India. Reprod Health Matters. 2012;20(39):21–30. 10.1016/S0968-8080(12)39604-3 22789079

[52] Barrow A. It’s like a rubber band. Assessing UNSCR 1325 as a gender mainstreaming process. Int Law Context. 2009;5(1):51–69.

[53] Garba AM, Bandali S. The Nigeria Independent Accountability Mechanism for maternal, newborn, and child health. Int J Gynecol Obstet. 2014;127(1):113–6. 10.1016/j.ijgo.2014.07.004 25179169

[54] Thaddeus S, Maine D. Too far to walk: Maternal mortality in context. Soc Sci Med. 1994;38(8):1091–110. 10.1016/0277-9536(94)90226-7 8042057

[55] Halloran B. Accountability ecosystems: directions of accountability and points of engagement. 2016;(June).

[56] Cleary SM, Molyneux S, Gilson L. Resources, attitudes and culture: An understanding of the factors that influence the functioning of accountability mechanisms in primary health care settings. BMC Health Serv Res. 2013;13(1):1–11. 23953492 10.1186/1472-6963-13-320 PMC3844434

[57] United Nations Office for the Coordination of Humanitarian Affairs. Global Humanitarian Overview 2018 [Internet]. 2017 [cited 2020 Feb 3]. Available from: https://interactive.unocha.org/publication/globalhumanitarianoverview/.

[58] Maru V. Access to justice and legal empowerment: A review of World Bank practice [Internet]. Washington D.C.; 2009. Available from: https://openknowledge.worldbank.org/bitstream/handle/10986/18102/518430NWP0Acce10Box342050B01PUBLIC1.pdf?sequence=1&isAllowed=y

[59] Goodwin L, Maru V. What Do We Know about Legal Empowerment? Mapping the Evidence. Hague J Rule Law. 2017;9(1):157–94.

[60] Cicely M, Rachael H, Kean S, Sushil B, Arti A, Costello A, et al Community participation for transformative action on women’s, children’s and adolescents’ health. Bull World Health Organ. 2016;94(5):376 10.2471/BLT.15.168492 27152056 PMC4857226

[61] Marston C, Renedo A, McGowan CR, Portela A. Effects of community participation on improving uptake of skilled care for maternal and newborn health: a systematic review. PLoS One. 2013;8(2):e55012 10.1371/journal.pone.0055012 23390509 PMC3563661

[62] George AS, Mehra V, Scott K, Sriram V. Community participation in health systems research: A systematic review assessing the state of research, the nature of interventions involved and the features of engagement with communities. PLoS One. 2015;10(10):1–25. 10.1371/journal.pone.0141091 PMC4619861 26496124

[63] Holland J, Schatz F. Macro evaluation of DFID’s policy frame for empowerment and accountability. Oxford: e-Pact; 2016.

[64] Westhorp G, Walker B, Rogers P, Overbeeke N, Ball D, Brice G. Enhancing community accountability, empowerment and education outcomes in low and middle-income countries: A realist review. 2014.

[65] Bukenya B, Hickey S, King S. Understanding the role of context in shaping social accountability interventions: towards an evidence-based approach. 2012.

[66] Van Belle S, Mayhew SH. What can we learn on public accountability from non-health disciplines: A meta-narrative review. BMJ Open. 2016;6(7). 10.1136/bmjopen-2015-010425 PMC4947774 27388347

[67] Transparency for Development. Transparency for Development Accountability for Health Policy Brief. 2019.

[68] Beck DC, Munro-Kramer ML, Lori JR. A scoping review on community mobilisation for maternal and child health in sub-Saharan Africa: Impact on empowerment. Glob Public Health. 2019;14(3):375–95. 10.1080/17441692.2018.1516228 30182808 PMC6459730

[69] Schuler SR, Islam F, Rottach E. Women’s empowerment revisited: a case study from Bangladesh. Dev Pract. 2010;20(7):840–54. 10.1080/09614524.2010.508108 20856695 PMC2941240

[70] Gram L, Morrison J, Skordis-Worrall J. Organising Concepts of ‘Women’s Empowerment’ for Measurement: A Typology. Soc Indic Res [Internet]. 2019;143(3):1349–76. Available from: 10.1007/s11205-018-2012-2 31231148 PMC6548747

[71] Scott K., Jessani N, Qiu M BS. Developing more participatory and accountable institutions for health: identifying health system research priorities for the Sustainable Development Goal-era. Heal Policy Planning,. 2018;1–13. 10.1093/heapol/czy079 PMC6263024 30247610

[72] Lopez Franco E, Shankland A. Guidelines for designing and monitoring social accountability interventions [Internet]. 2018 Available from: www.ids.ac.uk/publications.

[73] Shutt C, McGee R. Improving the Evaluability of INGO Empowerment and Accountability Programmes. 2013.

[74] Mcgowan C, Marston C, Steyn P, Boydell V, Mcgowan C, Marston C, et al International prospective register of systematic reviews Systematic review of study designs to measure effects of social accountability interventions in reproductive, maternal, newborn, child and adolescent health (RMNCAH) programs Citation Review qu. 2018;1–3.

[75] The Partnership for Maternal N and CH. 2018 Social Accountability Symposium for Women’s, Children’s and Adolescents’ Health: Bridging evidence, experience and practice [Internet]. 2018 [cited 2019 Sep 29]. p. 2–4. Available from: https://www.who.int/pmnch/media/news/2018/social-accountability-symposium/en/.

