# Supplementary material for: Social accountability for reproductive, maternal, newborn, child and adolescent health: A review of reviews
Source: PLoS One. 2020 Oct 9;15(10):e0238776. doi: 10.1371/journal.pone.0238776 (PMC7546481; doi:10.1371/journal.pone.0238776)
Supplement: S1 Annex — (DOCX) [file pone.0238776.s003.docx]

**Annex 1: PubMed search strategy**

Search conducted on 20/05/19

"social accountab*" OR Social Responsibility [MeSH] OR Community Participation [MeSH] OR accountab* OR “collective action” OR “community action” OR "social mobilisation” OR "social mobilization" OR “community mobilisation” OR "community mobilization" OR “social movement*” OR “community movement*” OR “participatory budgeting” OR “public expenditure tracking” OR “citizen charter*” OR “public hearing*” OR “citizen report card*” OR “social audit*” OR “health committee*” OR “community scorecard*” OR "community score card" OR “complaint mechanism*” OR “social protest*” OR "participatory governance" OR "patient advocacy" OR "community monitoring" OR "community participation" OR "civil society" OR "social activism"

 AND

Systematic review OR synthes* OR mapping OR review* OR systemat*

AND

Reproductive Health [MeSH] OR Sexual health [MeSH] OR Maternal Health [MeSH] OR "newborn health" OR Adolescent Health [MeSH] OR neonat* OR Child Health [MeSH] OR Contraception OR HIV OR Sexually Transmitted Diseases [MeSH] OR abortion* OR "cervical cancer" OR "gender-based violence" OR "intimate partner violence" OR "violence against women" OR "female genital cutting" OR "female genital mutilation" OR “family planning" OR STI

10890 results

Filters for “humans” and “reviews” selected

650 results
