# Supplementary material for: Social accountability for reproductive, maternal, newborn, child and adolescent health: A review of reviews
Source: PLoS One. 2020 Oct 9;15(10):e0238776. doi: 10.1371/journal.pone.0238776 (PMC7546481; doi:10.1371/journal.pone.0238776)
Supplement: S2 Annex — (DOCX) [file pone.0238776.s004.docx]

**Annex 2: Articles excluded at full text review**

Altman L, Kuhlmann AK, Galavotti C. 2015. Understanding the black box: a systematic review of the measurement of the community mobilization process in evaluations of interventions targeting sexual, reproductive, and maternal health. *Eval Program Plann.* **49:**86-97. doi: 10.1016/j.evalprogplan.2014.11.010.

Beck DC, Munro-Kramer ML, Lori JR. 2018. A scoping review on community mobilisation for maternal and child health in sub-Saharan Africa: Impact on empowerment. *Glob Public Health.* 5:1-21. doi: 10.1080/17441692.2018.1516228.

Busza J, Walker D, Hairston A, Gable A, Pitter C, Lee S, Katirayi L, Simiyu R, Mpofu D. 2012. Community-based approaches for prevention of mother to child transmission in resource-poor settings: a social ecological review. *J Int AIDS Soc.* **11**:15 Suppl 2:17373. doi: 10.7448/IAS.15.4.17373.

Cornish F, Priego-Hernandez J, Campbell C, Mburu G, McLean S. 2014. The impact of community mobilisation on HIV prevention in middle and low income countries: a systematic review and critique. *AIDS Behav.* **18(11):**2110-34. doi: 10.1007/s10461-014-0748-5.

Ferguson L, Halliday E. 2013. Participation and human rights: Impact on women's and children's health. What does the literature tell us? In: Bustreo F, Hunt P, Gruskin S, Eide A, L M, S R, et al., editors. Women's and children's health: Evidence of impact of human rights. Geneva: World Health Organixation. p. 140.

George A, Branchini C, Portela A. 2015. Do Interventions that Promote Awareness of Rights Increase Use of Maternity Care Services? A Systematic Review. *PLoS One.* **7;10(10):**e0138116. doi: 10.1371/journal.pone.0138116.

Gulaid A, Kiragu K. 2012. Lessons learnt from promising practices in community engagement for the elimination of new HIV infections in children by 2015 and keeping their mothers alive: summary of a desk review. *J Int AIDS Soc.* **11;15 Suppl 2**:17390. Doi: 10.7448/IAS.15.4.17390.

Hoffman, KD. 2014. The Role of Social Accountability in Improving Health Outcomes: Overview and Analysis of Selected International NGO Experiences to Advance the Field. Washington, DC: CORE Group.

Hurst TE, Semrau K, Patna M, Gawande A, Hirschhorn LR. 2015. Demand-side interventions for maternal care: evidence of more use, not better outcomes. *BMC Pregnancy Childbirth.* **13;15:**297. doi: 10.1186/s12884-015-0727-5.

Kraft JM, Wilkins KG, Morales GJ, WidyonoM , Middlestadt SE. 2014. An Evidence Review of Gender-Integrated Interventions in Reproductive and Maternal-Child Health. [*J Health Commun.*](https://www.ncbi.nlm.nih.gov/pubmed/25207450)  **19 Suppl 1:**122-41. doi: 10.1080/10810730.2014.918216.

Lawn JE, Kinney M, Lee AC, Chopra M, Donnay F, Paul VK, Bhutta ZA, Bateman M, Darmstadt GL. 2009. Reducing intrapartum-related deaths and disability: can the health system deliver? *Int J Gynaecol Obstet.* **107 Suppl 1**:S123-40, S140-2. doi: 10.1016/j.ijgo.2009.07.021.

Lee AC, Lawn JE, Cousens S, Kumar V, Osrin D, Bhutta ZA, Wall SN, Nandakumar AK, Syed U, Darmstadt GL. 2009. Linking families and facilities for care at birth: what works to avert intrapartum-related deaths? *Int J Gynaecol Obstet.* **107 Suppl 1**:S65-85, S86-8. doi: 10.1016/j.ijgo.2009.07.012.

Marston C, Renedo A, McGowan CR, Portela A. 2013. Effects of community participation on improving uptake of skilled care for maternal and newborn health: a systematic review. *PLoS One.* **8(2):**e55012. doi: 10.1371/journal.pone.0055012.

Moore L, Chersich MF, Steen R, Reza-Paul S, Dhana A, Vuylsteke B, Lafort Y, Scorgie F. 2014. Community empowerment and involvement of female sex workers in targeted sexual and reproductive health interventions in Africa: a systematic review. *Global Health.* **10;10:**47. doi: 10.1186/1744-8603-10-47.

Pilkington G, Panday S, Khatib MN, Kotas E, Hill RA, Simkhada P, Jones L. 2018. The effectiveness of community engagement and participation approaches in low and middle income countries: a review of systematic reviews with particular reference to the countries of South Asia. London: EPPI Centre, Social Science Research Unit, Institute of Education, University of London.

Prost A, Colbourn T, Seward N, Azad K, Coomarasamy A, Copas A, Houweling TA, Fottrell E, Kuddus A, Lewycka S, MacArthur C, Manandhar D, Morrison J, Mwansambo C, Nair N, Nambiar B, Osrin D, Pagel C, Phiri T, Pulkki-Brännström AM, Rosato M, Skordis-Worrall J, Saville N, More NS, Shrestha B, Tripathy P, Wilson A, Costello A. 2013. Women's groups practising participatory learning and action to improve maternal and newborn health in low-resource settings: a systematic review and meta-analysis. *Lancet.* **18;381(9879):**1736-46. doi: 10.1016/S0140-6736(13)60685-6.

Sebert Kuhlmann AK, Altman L, Galavotti C. 2016. The importance of community mobilization in interventions to improve sexual, reproductive, and maternal health outcomes: A review of the evidence. *Health Care Women Int.* **37(10):**1028-66. doi: 10.1080/07399332.2016.1141911.

Shah, NM, Brieger WR, Peters DH. 2011. Can interventions improve health services from informal private providers in low and middle-income countries? A comprehensive review of the literature. *Health Policy Plan.* **26(4):**275-87. doi: 10.1093/heapol/czq074. Epub 2010 Nov 19.

Steyn PS, Cordero JP, Gichangi P, Smit JA, Nkole T, Kiarie J, Temmerman M. 2016. Participatory approaches involving community and healthcare providers in family planning/contraceptive information and service provision: a scoping review. *Reprod Health.* **13(1):**88. doi: 10.1186/s12978-016-0198-9.

Wallerstein N. 2006. What is the evidence on effectiveness of empowerment to improve health? Copenhagen: WHO Regional Office for Europe.

Wekesah FM, Mbada CE, Muula AS, Kabiru CW, Muthuri SK, Izugbara CO. 2016. Effective non-drug interventions for improving outcomes and quality of maternal health care in sub-Saharan Africa: a systematic review. *Syst Rev.* **15;5(1):**137. doi: 10.1186/s13643-016-0305-6.
