# Supplementary material for: Social accountability for reproductive, maternal, newborn, child and adolescent health: A review of reviews
Source: PLoS One. 2020 Oct 9;15(10):e0238776. doi: 10.1371/journal.pone.0238776 (PMC7546481; doi:10.1371/journal.pone.0238776)
Supplement: S3 Annex — (DOCX) [file pone.0238776.s005.docx]

**Annex 3: Quality assessment checklist**

| **Review quality assessment checklist** | | | | |
| --- | --- | --- | --- | --- |
| **Title of paper** | |  | | |
| **Author/s** | |  | | |
| **Name of assessor** | |  | | |
| **Methods used to identify, include and critically appraise studies** | | | | |
| **Question** | | **Key considerations** | **Score** | **Notes** |
| A1. Was the search for evidence reasonably comprehensive? | | - Language bias avoided - No restriction of inclusion based on publication status - Relevant databases searched, including grey literature - Reference lists in included articles checked - Authors/experts contacted | Yes  No  Partially  Unsure / Can’t tell |  |
| A2. Does the review cover an appropriate time period? | | - Is the search period comprehensive enough that relevant literature is unlikely to be omitted? | Yes  No  Partially  Unsure / Can’t tell |  |
| A3. Was bias in the selection of articles avoided? | | - Independent screening of full text by at least two reviewers - List of included studies provided - List of excluded studies provided | Yes  No  Partially  Unsure / Can’t tell |  |
| A4. Were the criteria used for deciding which studies to include in the review reported? | | - Do the authors specify the types of studies, participants/settings / population, interventions, outcomes? | Yes  No  Partially  Unsure / Can’t tell |  |
| A5. Were the primary studies included in the review well designed and conducted? | | - How were participants and settings selected? - How were data were collected / analysed? - Was potential bias adequately addressed? | Yes  No  Partially  Unsure / Can’t tell |  |
| A6. Did the authors use appropriate criteria to assess the quality and risk of bias in analysing the studies that are included? | | - Did the authors use appropriate criteria to assess the quality and risk of bias in analysing the studies that are included? - Were the criteria used for assessing the quality/ risk of bias reported? | Yes  No  Partially  Unsure / Can’t tell |  |
| **Overall assessment of quality of methods used to identify, include and critically appraise studies**  *(Low confidence = 0 to 2 yes results; medium confidence = 2 to 4 yes results; high confidence = 5 to 6 yes results)* | | | | Low confidence  Medium confidence  High confidence  Unclear |
| **Methods used to analyse findings** | | | | |
| **Question** | **Key considerations** | | **Score** | **Notes** |
| B1. Were the characteristics and results of the included studies reliably reported? | - Independent data extraction by at least two reviewers? - A table or summary of the characteristics of the participants, interventions and outcomes for the included studies. - A table or summary of the results of all the included studies. | | Yes  No  Partially |  |
| B2. Was the chosen method of data analysis clearly described and appropriate for the review question? | - Was the method used to analyse the findings of the included studies clear? - Was the method of data analysis appropriate for the included study designs and review question? - Were the findings of the relevant studies combined (or not combined) appropriately relative to the primary question and the available data? - For quantitative studies: how were the studies weighted in the analysis? Did the review address unit of analysis errors? Consider also methods for calculating effect sizes. | | Yes  No  Partially |  |
| B3. Did the review describe the extent of heterogeneity? | - Did the review discuss the extent to which there were important differences in the results of the included studies? - Did the review examine specific factors which might explain differences in the results of the included studies? - Was a sensible method used to explore the extent to which key factors explained heterogeneity? | | Yes  No  Partially |  |
| B4. Does the review report potential bias appropriately? | - The review makes clear which evidence is subject to low risk of bias and which is likely to be biased, and does so appropriately - Where studies of differing risk of bias are included, results are reported and analysed separately by risk of bias status. | | Yes  No  Partially |  |
| B5. Is there a clear and cogent connection between the studies and the review findings? | - Are any of the data are ambiguous? - Do the data support or contradict the review findings? - Were factors that the review authors considered as likely explanatory factors clearly described? | | Yes  No  Partially  Unsure / Can’t tell |  |
| B6. Are the data supporting the review findings of sufficient richness and quantity? | - Are the data sufficiently rich and detailed? - Do they come from an adequate number of studies / participants? | | Yes  No  Unsure / Can’t tell |  |
| **Overall assessment of quality of methods used to analyse findings**  *(Low confidence = 0 to 2 yes results; medium confidence = 3 to 5 yes results; high confidence = 6 to 7 yes results)* | | | | Low confidence  Medium confidence  High confidence  Unclear |
| **Overall assessment** | | | | |
| **Question** | **Key considerations** | | **Score** | **Notes** |
| C1. Are there any other aspects of the review not mentioned before which lead you to question the results? | - Additional methodological concerns - Robustness of findings - Problems with interpretation of data - Conflicts of interest (of the review authors or for included studies) | | Yes  No |  |
| C2. Are there any mitigating factors which should be taken into account in determining the review’s reliability? | - Limitations acknowledged - No strong policy conclusions drawn (including in abstract/ summary) - Any other factors | | Yes  No |  |
| **Overall assessment of quality and reliability of the review**  *(Make an overall assessment of confidence in the review, by combining results from section A and B, and considering the additional points in Section C.)* | | | | Low confidence  Medium confidence  High confidence  Unclear |
